# Syntheses and Biological Studies of Cu(II) Complexes Bearing Bis(pyrazol-1-yl)- and Bis(triazol-1-yl)-acetato Heteroscorpionate Ligands

**DOI:** 10.3390/molecules24091761

**Published:** 2019-05-07

**Authors:** Maura Pellei, Valentina Gandin, Luciano Marchiò, Cristina Marzano, Luca Bagnarelli, Carlo Santini

**Affiliations:** 1 School of Science and Technology, Chemistry Division, University of Camerino, via S. Agostino 1, 62032 Camerino, Macerata, Italy; luca.bagnarelli@unicam.it (L.B.); carlo.santini@unicam.it (C.S.); 2 Department of Pharmaceutical and Pharmacological Sciences, University of Padova, via Marzolo 5, 35131 Padova, Italy; cristina.marzano@unipd.it; 3 Department of Chemistry, Life Science, and Environmental Sustainability, University of Parma, Parco Area delle Scienze 17A, 43124 Parma, Italy; luciano.marchio@unipr.it

**Keywords:** copper, poly(azolyl)acetate ligands, X-ray, spectroscopy, cytotoxicity

## Abstract

Copper(II) complexes of bis(pyrazol-1-yl)- and bis(triazol-1-yl)-acetate heteroscorpionate ligands have been synthesized. The copper(II) complexes [HC(COOH)(pz^Me2^)_2_]Cu[HC(COO)(pz^Me2^)_2_]·ClO_4_, [HC(COOH)(pz)_2_]_2_Cu(ClO_4_)_2_ (pz^Me2^ = 3,5-dimethylpyrazole; pz = pyrazole) were prepared by the reaction of Cu(ClO_4_)_2_·6H_2_O with bis(3,5-dimethylpyrazol-1-yl)acetic acid (HC(COOH)(pz^Me2^)_2_) and bis(pyrazol-1-yl)acetic acid (HC(COOH)(pz)_2_) ligands in ethanol solution. The copper(II) complex [HC(COOH)(tz)_2_]_2_Cu(ClO_4_)_2_·CH_3_OH (tz = 1,2,4-triazole) was prepared by the reaction of Cu(ClO_4_)_2_·6H_2_O with bis(1,2,4-triazol-1-yl)acetic acid (HC(COOH)(tz)_2_) ligand in methanol solution. The synthesized Cu(II) complexes, as well as the corresponding uncoordinated ligands, were evaluated for their cytotoxic activity in monolayer and 3D spheroid cancer cell cultures with different Pt(II)-sensitivity. The results showed that [HC(COOH)(pz^Me2^)_2_]Cu[HC(COO)(pz^Me2^)_2_]·ClO_4_ was active against cancer cell lines derived from solid tumors at low IC_50_ and this effect was retained in the spheroid model. Structure and ultra-structure changes of treated cancer cells analyzed by Transmission Electron Microscopy (TEM) highlighted the induction of a cytoplasmic vacuolization, thus suggesting paraptotic-like cancer cell death triggering.

## 1. Introduction

Copper complexes have been synthesized and highlighted as anticancer agents on the assumption that endogenous metal ions are less toxic to normal cells than non-endogenous metals [1,2,3]. Recently, copper complexes showed promising in vitro and in vivo results as anticancer agents [4,5,6,7,8,9,10,11,12,13], due to the raised need for copper by cancer tissues and the established role of copper for tumor growth, angiogenesis and metastasis [5,14,15,16,17]. Furthermore, there is increasing evidence that the copper compounds act as antitumor drugs with a different mechanism of action able to circumvent the problems encountered by cisplatin [18], displaying a broader spectrum of activities and lower toxicity [19,20,21,22], and representing potential worthwhile alternatives to Pt-based drugs [23,24].

Among the well-known scorpionates [2,25,26], bis(azol-1-yl)acetate heteroscorpionate ligands of general formula [HC(COOH)(az)_2_] (az = *N*-heterocyclic ring), first synthesized in 1999 by A. Otero and coworkers [27] and in 2001 by N. Burzlaff and coworkers [28,29], have recently attracted considerable attention, and their coordination chemistry towards main group and transition metals has been extensively studied [30,31,32].

Among them, complexes containing bis(pyrazol-1-yl)carboxylic acids are especially of interest, due to their κ^3^-*N*,*N*,*O* tripodal coordination behavior, as metalloenzyme models relevant for biochemistry [28,33,34,35,36,37,38,39,40,41,42] and as starting materials to yield bifunctional ligands [43,44,45,46]. In the nearly 15 years, the facially coordinating bis(pyazol-1-yl)acetate ligands, typically substituted at the 3,5-positions of the pyrazolyl rings, have been used to synthesize several structurally characterized copper(II) complexes [34,47,48,49,50,51,52,53,54,55,56,57,58]. Many of these coordination compounds were studied for their unique structural, electrochemical, and catalytic properties; however, to our knowledge, biological studies on the anticancer properties of bis(pyrazolyl)acetate copper(II) complexes are unknown.

During the last decades, in our quest to find suitable ligands in the development of metal-based anticancer agents [1], we designed and synthesized new bis(azol-1-yl)carboxylate ligands with pyrazole, triazole, imidazole or pyridine scaffolds [59,60,61,62,63]. Bis(azol-1-yl)carboxylic acids are useful starting materials to yield neutral heteroscorpionate ligands functionalized with acetamide or thioacetamide groups [64,65,66,67,68,69]. In recent works, they have been conjugated with glucosamine, 5-nitroimidazole and a *N*-methyl-d-aspartate (NMDA) receptor antagonist and the related Cu(I) and Cu(II) complexes have been investigated for their cytotoxic activity towards several human tumor cell lines [43,44,45,46].

The aim of the present research is to deepen the study of copper(II) complexes based on bis(azol-1-yl)-acetate heteroscorpionate ligands as anticancer agents. For such a purpose, here we report the synthesis of Cu(II) complexes of the previously synthesized bis(pyrazol-1-yl)- and bis(1,2,4-triazol-1-yl)-acetate ligands [59]. All complexes were assessed for their cytotoxic potential in monolayer and 3D spheroid cancer cell cultures with different Pt(II)-sensitivity. Moreover, by means of biochemical assays and Transmission Electron Microscopy (TEM) analyses, mechanistic properties of the most promising derivative were investigated.

## 2. Experimental Section

### 2.1. Materials and Instruments

All syntheses and handling were carried out under an atmosphere of dry oxygen-free dinitrogen, using standard Schlenk techniques. All solvents were dried, degassed and distilled prior to use. Elemental analyses (C,H,N,S) were performed with a Fisons Instruments EA-1108 CHNS-O Elemental Analyzer (Thermo Fisher Scientific Inc., Waltham, MA, USA). Melting points were taken on an SMP3 Stuart Scientific Instrument (Bibby Sterilin Ltd., London, UK). IR spectra were recorded from 4000 to 400 cm^−1^ on a PerkinElmer Frontier FT-IR instrument (PerkinElmer Inc., Waltham, MA, USA), equipped with single reflection universal diamond ATR top-plate. IR annotations used: br = broad, m = medium, s = strong, sbr = strong broad, sh = shoulder, vbr = very broad, w = weak, wbr = weak broad. Electrospray mass spectra (ESI-MS) were obtained in positive- (ESI(+)MS) or negative-ion (ESI(−)MS) mode on an Agilent Technologies Series 1100 LC/MSD Mass Spectrometer (Agilent Technologies Inc, Santa Clara, CA, USA), using a methanol or acetonitrile mobile phase. The compounds were added to reagent grade methanol to give approximately 0.1 mM solutions, injected (1 μL) into the spectrometer via a Hewlett Packard 1090 Series II UV-Visible HPLC system (Agilent Technologies Inc, Santa Clara, CA, USA) fitted with an autosampler. The pump delivered the solutions to the mass spectrometer source at a flow rate of 300 mL min^−1^, and nitrogen was employed both as a drying and nebulizing gas. Capillary voltages were typically 4000 V and 3500 V for the ESI(+)MS and ESI(−)MS mode, respectively. Confirmation of all major species in this ESI-MS study was supported by comparison of the observed and predicted isotope distribution patterns, the latter calculated using the IsoPro 3.1 computer program (T-Tech Inc., Norcross, GA, USA).

### 2.2. Synthesis

All reagents were purchased from Sigma-Aldrich (St. Louis, MO, USA) and used without further purification. The ligands [HC(COOH)(pz^Me2^)_2_] [28], [HC(COOH)(pz)_2_] [29] and [HC(COOH)(tz)_2_] [59] were prepared by methods in cited literature.

Although we experienced no difficulties with the perchlorate salts described, these compounds should be regarded as potentially explosive and handled according to Ref. [70].

#### 2.2.1. Synthesis of [HC(COOH)(pz^Me2^)_2_]Cu[HC(COO)(pz^Me2^)_2_]·ClO_4_, (**1**)

An ethanol solution (25 mL) of Cu(ClO_4_)_2_·6H_2_O (0.137 g, 0.4 mmol) was added to an ethanol solution (25 mL) of [HC(COOH)(pz^Me2^)_2_] (0.184 g, 0.8 mmol). After the addition, the reaction mixture was stirred at room temperature for 24 h to obtain a blue precipitate which was filtered off and dried to constant weight to give complex **1** in 62% yield. By dissolving the crude complex (**1**) in CH_3_CN solution and by slow evaporation of the solution, single crystals of **1** suitable for X-ray diffraction analysis (Table 1) were obtained. M.p. 211–215 °C dec. IR (cm^−1^): 3459vbr (OH); 3139w, 2972w (CH); 1705br, 1667sh (ν_asym_ COO); 1560m (C=N_pz_); 1464m, 1419m; 1390m, 1377m (ν_sym_ COO); 1312m, 1246m, 1222br; 1081s, 1050sh (ClO_4_); 987m, 939m, 909m, 883m, 815m, 804m, 774m, 743m, 712m, 694s, 668m, 655m. ESIMS (major positive-ions, CH_3_CN), *m*/*z* (%): 558 (100) [{HC(COOH)(pz^Me2^)_2_}Cu{HC(COO)(pz^Me2^)_2_}]^+^, 869 (30) [{HC(COO)(pz^Me2^)_2_}_3_Cu_2_]^+^, 1117 (10) [{HC(COO)(pz^Me2^)_2_}_4_Cu_2_ + H]^+^. ESIMS (major negative-ions, CH_3_OH), *m*/*z* (%): 99 (100) [ClO_4_]^−^. Calcd. for C_24_H_32_ClCuN_8_O_8_: C, 43.77; H, 4.74; N, 17.02%. Found: C, 43.80; H, 4.77; N, 16.75%.

#### 2.2.2. Synthesis of [HC(COOH)(pz)_2_]_2_Cu(ClO_4_)_2_, (**2**)

An ethanol solution (25 mL) of Cu(ClO_4_)_2_·6H_2_O (0.185 g, 0.5 mmol) was added to an ethanol solution (25 mL) of [HC(COOH)(pz)_2_] (0.192 g, 1.0 mmol). After the addition, the reaction mixture was stirred at room temperature for 24 h to obtain a blue precipitate which was filtered off and dried to constant weight to give complex **2** in 58% yield. M.p. 206–210 °C dec. IR (cm^−1^): 3557wbr (OH); 3133w, 3117w, 3016w (CH); 1736br (ν_asym_ COO); 1515m (C=N_pz_); 1451m, 1409s; 1372m (ν_sym_ COO); 1348w, 1284w, 1218br; 1085s, 1067s (ClO_4_); 996m, 928m, 906br, 861m, 847m, 790m, 765s, 712sbr, 677s. ESIMS (major positive-ions, CH_3_CN), *m*/*z* (%): 446 (100) [{HC(COO)(pz)_2_}_2_Cu + H]^+^. ESIMS (major negative-ions, CH_3_CN), *m*/*z* (%): 99 (100) [ClO_4_]^−^. Calcd. for C_16_H_16_Cl_2_CuN_8_O_12_: C, 29.71; H, 2.49; N, 17.32%. Found: C, 30.14; H, 2.15; N, 16.96%.

#### 2.2.3. Synthesis of [HC(COOH)(tz)_2_]_2_Cu(ClO_4_)_2_·CH_3_OH, (**3**)

A methanol solution (40 mL) of Cu(ClO_4_)_2_·6H_2_O (0.185 g, 0.5 mmol) was added to a methanol solution (40 mL) of [HC(COOH)(tz)_2_] (0.194 g, 1.0 mmol). After the addition, the reaction mixture was stirred at room temperature for 24 h to obtain a pale blue precipitate which was filtered off and dried to constant weight to give complex **3** in 51% yield. M.p. 195–199 °C dec. IR (cm^−1^): 3446br (OH); 3134m, 2977w (CH); 1664br (ν_asym_ COO); 1528m (C=N_pz_); 1456w; 1365m (ν_sym_ COO); 1283m, 1208m; 1125s, 1083sbr (ClO_4_); 1021m, 995m, 931w, 889m, 832m, 760s, 670s. ESIMS (major positive-ions, DMSO/CH_3_CN), *m*/*z* (%): 290 (30) [{HC(COO)(tz)_2_}Cu(CH_3_OH)]^+^. ESIMS (major negative-ions, DMSO/CH_3_CN), *m*/*z* (%): 99 (100) [ClO_4_]^−^, 149 (10) [HC(tz)_2_]^−^, 193 (10) [HC(COO)(tz)_2_]^−^, 221 (100) [Na(ClO_4_)_2_]^−^, 360 (60) [Cu(ClO_4_)_3_]^−^. Calcd. for C_13_H_16_Cl_2_CuN_12_O_13_: C, 22.87; H, 2.36; N, 24.62%. Found: C, 22.49; H, 2.55; N, 24.14%.

### 2.3. X-ray Crystallography

A summary of data collection and structure refinement for [HC(COOH)(pz^Me2^)_2_]Cu[HC(COO)(pz^Me2^)_2_]·ClO_4_, (**1**) is reported in Table 1. Single crystal data were collected with a Bruker diffractometer (Karlsruhe, Germany), model Smart equipped with a Breeze area detector, Mo Kα:λ = 0.71073 Å. The intensity data were integrated from several series of exposures frames (0.3° width) covering the sphere of reciprocal space [71]. Absorption correction were applied using the program SADABS [72]. The structures were solved by direct methods using SIR2004 [73]. Fourier analysis and refinement were performed by the full-matrix least-squares methods based on F^2^ implemented in SHELXL-2014 [74]. Within the [HC(COOH)(pz^Me2^)_2_]Cu[HC(COO)(pz^Me2^)_2_]^+^ complex cation, the carboxylate functions of the ligands were found disordered in two positions, which were refined with site occupancy factors of 0.5 each. The perchlorate anion was located into a spherical structural site and it was found disordered in four positions having 0.25 site occupancy factors each. Graphical material was prepared with the Mercury program [75]. CCDC 1905998 contains the supplementary crystallographic data for this paper.

### 2.4. Experiments with Human Cells

Complexes **1** and **2,** uncoordinated ligands, cisplatin and oxaliplatin were solubilized in 0.9% NaCl solution. Complex **3** was solubilized in stock DMSO solutions (10 mg/mL) and added to the culture medium to a final solvent concentration of 0.5%, which had no effects on cell viability. MTT (3-(4,5-dimethylthiazol-2-yl)-2,5-diphenyltetrazolium bromide), fluorogenic peptide proteasomal substrates (*N*-Suc-Leu-Leu-Val-Tyr-7-amido-4-methylcoumarin (AMC), Boc-Gln-Ala-Arg-AMC, and *Z*-Leu-Leu-Glu-AMC) cisplatin and oxaliplatin were obtained from Sigma Chemical Co, St. Louis, MO, USA.

#### 2.4.1. Cell Cultures

Human lung (A549), colon (HCT-15 and LoVo) and breast (MCF-7) carcinoma cell lines were obtained from American Type Culture Collection (ATCC, Rockville, MD, USA). Human pancreatic BxPC3 carcinoma cells were obtained from European Collection of Cell Culture (ECACC, Salisbury, UK). Human ovarian 2008 cancer cells were kindly provided by Prof. G. Marverti (Dept. of Biomedical Science of Modena University, Modena, Italy). Human squamous cervical A431 carcinoma cells were kindly provided by Prof. F. Zunino (Division of Experimental Oncology B, Istituto Nazionale dei Tumori, Milan, Italy). The LoVo-OXP cells were obtained as previously described [76]. Cell lines were maintained in culture in the logarithmic phase at 37 °C in a 5% carbon dioxide atmosphere using the following media added of 10% fetal calf serum (Euroclone, Milan, Italy), antibiotics (50 units/mL penicillin and 50 μg/mL streptomycin), and 2 mM l-glutamine: (i) RPMI-1640 medium (Euroclone) for HCT-15, A431, MCF-7, BxPC3 and 2008 cells; (ii) F-12 HAM’S (Sigma Chemical Co.) for A549, LoVo and LoVo-OXP cells.

#### 2.4.2. Spheroid Cultures

Spheroid cultures were obtained by seeding 2.5 × 10^3^ HCT-15 cells/well in round bottom non-tissue culture treated 96 well-plate (Greiner Bio-one, Kremsmünster, Austria) in phenol red free RPMI-1640 medium (Sigma Chemical Co.), containing 10% FCS and supplemented with 20% methyl cellulose stock solution. 

#### 2.4.3. Cytotoxicity Assays

##### MTT Assay

The growth inhibitory effect towards 2D tumor cell lines was evaluated by means of MTT assay as previously described [44,77]. IC_50_ values were calculated by four parameters logistic (4-PL) model. All the values are the means ± SD of not less than five measurements starting from three different cell cultures.

##### Acid Phosphatase (APH) Assay

An APH modified assay was used for determining cell viability in 3D spheroids, as previously described [5]. IC_50_ values were calculated with a four parameter logistic (4-PL) model. All the values are the means ± SD of not less than four independent experiments.

#### 2.4.4. Cellular Uptake

LoVo cells (2.5 × 10^6^) were seeded in 75 cm^2^ flasks in growth medium (20 mL). After overnight incubation, the medium was replaced, and the cells were treated with tested compounds for 24 h. Cell monolayers were then processed and mineralized as previously described [44]. The sample was analyzed for copper by using a Varian AA Duo graphite furnace atomic absorption spectrometer (Varian, Palo Alto, CA, USA) at the wavelength of 324 nm. The calibration curve was obtained using known concentrations of standard solutions purchased from Sigma Chemical Co.

#### 2.4.5. ROS Production

The production of ROS was measured in LoVo cells (10^4^ per well) grown for 24 h in a 96-well plate in RPMI medium without phenol red. Samples were loaded with 10 μM 5-(and-6)-chloromethyl-2′,7′-dichlorodihydrofluorescein diacetate acetyl ester (CM-H_2_DCFDA) (Molecular Probes-Invitrogen, Eugene, OR, USA) for 25 min, in the dark. Afterward, each well was washed with PBS and incubated with tested complexes. The fluorescence intensity in each well was assessed by means of a Fluoroskan Ascent FL (Labsystem, Vantaa, Finland) plate reader (ex: 485 nm; em: 527 nm). Antimycin (3 μM, Sigma), a well-known inhibitor of the electron transport chain at Complex III level, was used as reference compound.

#### 2.4.6. Proteasome Inhibition

The purified rabbit 26S proteasome (Sigma Aldrich) was incubated for 60 min at 37 °C in assay buffer (50 mM Tris-HCl, pH 7.5, 250 mM sucrose, 5 mM MgCl_2_, 1 mM DTT, and 0.5 mM EDTA), in the presence of 15 µM of **1**–**3** or 10 µM Lactacystin. Afterwards, fluorogenic peptides were added and substrate hydrolysis was measured after 30 min by monitoring spectrofluorometrically the release of AMC (excitation at 370 nm, emission at 460 nm).

#### 2.4.7. Transmission Electron Microscopy Analyses

Approximately 10^6^ LoVo cells were seeded in 24-well plates and, after 24 h incubation, treated with **1** and incubated further 24 h. Samples were then washed with cold PBS, harvested and fixed with 1.5% glutaraldehyde buffer with 0.2 M sodium cacodylate, pH 7.4. After washing with buffer and post-fixation with 1% OsO_4_ in 0.2 M cacodylate buffer, samples were desiccated and embedded in epoxy resin (Epon Araldite). Sagittal serial sections of about 1 μm were counterstained with toluidine blue; subsequently, sections of 90 nm were stained with uranyl acetate and lead citrate.

Micrographs were taken with a Hitachi H-600 electron microscope (Hitachi, Tokyo, Japan) at 75 kV. All photos were elaborated in Corel Draw 11 (Corel Corporation, Ottawa, ON, Canada).

#### 2.4.8. Statistical Analysis

All values are the means ± SD of no less than three measurements. Multiple comparisons were eventually made by ANOVA followed by the Tukey–Kramer multiple comparison test (* *P* < 0.1, ** *P* < 0.01), using GraphPad software 7 (GraphPad Software Inc., San Diego, CA, USA).

## 3. Results and Discussion

### 3.1. Synthesis

**Figure 1:**
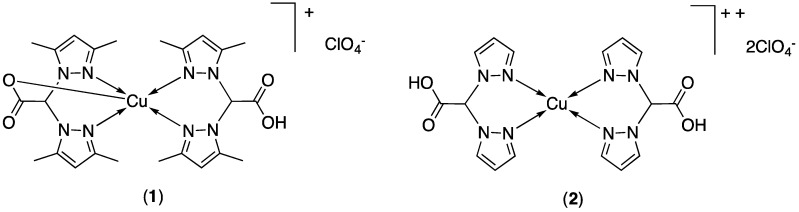
Structure of compounds **1** and **2**.

The ligands HC(COOH)(pz^Me2^)_2_ [28], HC(COOH)(pz)_2_ [29] and [HC(COOH)(tz)_2_] [59] were prepared by a method described in the literature and were fully characterized. The related copper(II) complexes [HC(COOH)(pz^Me2^)_2_]Cu[HC(COO)(pz^Me2^)_2_]·ClO_4_ (**1**) and [HC(COOH)(pz)_2_]_2_Cu(ClO_4_)_2_ (**2**) have been prepared from the reaction of Cu(ClO_4_)_2_·6H_2_O with [HC(COOH)(pz^Me2^)_2_] and [HC(COOH)(pz)_2_], respectively, in ethanol solution at room temperature (Figure 1). The compounds **1** and **2** are soluble in methanol, DMSO and acetonitrile are air stable even as solutions; in water, **1** is slightly soluble and **2** is soluble. Both are insoluble in diethyl ether, n-hexane, dichloromethane and chloroform. The authenticity of **1** and **2** was confirmed by elemental analysis, IR spectroscopy and Electrospray mass spectra. The infrared spectra showed all the bands required by the presence of the scorpionate donors. Medium broad absorptions at 1705 and 1667 cm^−1^, were observed in the spectrum of compound **1**, due to the carbonylic asymmetric stretching. They were shifted with respect to the same absorption observed for the free ligand of [HC(COOH)(pz^Me2^)_2_] (1740 cm^−1^) and were in accordance with the presence of the uncoordinated protonated carboxylic group and coordinated to the metal center carboxylate ligand, respectively [34,47,48,50,58]. A broad absorption at 1736 cm^−1^ was present in the spectrum of compound **2**, due to the carbonylic asymmetric stretching of the COOH groups in the same range observed for the free ligand [HC(COOH)(pz)_2_] (1722 cm^−1^). The difference between asymmetric and symmetric frequencies Δ[(ν_asym_ COO) − (ν_sym_ COO)] was 364 cm^−1^, which supports the non-coordinating mode of the two carboxylic groups. In the spectra of compounds **1** and **2** weak absorptions due to the CH stretchings have been observed in the range 2972–3139 cm^−1^, while medium bands in the range 1515–1560 cm^−1^ were attributable to the ν(C=N_pz_) stretching vibrations. Strong peaks in the range 1050–1085 cm^−1^ confirmed the presence of the ClO_4_^−^ groups as counter-ion species.

ESI-MS spectroscopy was used to probe the existence of complexes in solution. Both positive- and negative-ion spectra of complexes **1** and **2** in acetonitrile solutions were recorded at low voltage to minimize the dissociation and to transport the most of the analyte to the mass spectrometer as intact molecular species [78]. In acetonitrile solution, the positive-ion spectrum of compound **1** was dominated by the molecular ion [HC(COOH)(pz^Me2^)_2_]Cu[HC(COO)(pz^Me2^)_2_]^+^ (*m/z* 558, 100%) along with clusters at *m*/*z* 869 ([{HC(COO)(pz^Me2^)_2_}_3_Cu_2_]^+^, 30%) and 1117 ([{HC(COO)(pz^Me2^)_2_}_4_Cu_2_ + H]^+^, 10%). Analogously, the positive-ion spectrum of compound **2** was dominated by the aggregate [{HC(COO)(pz)_2_}_2_Cu + H]^+^ at *m/z* 446 (100%). The ESI(−)MS spectra of **1** and **2** displayed a metal free peak at *m*/*z* 99 (100%), attributable to the [ClO_4_]^−^ ion.

**Figure 2:**
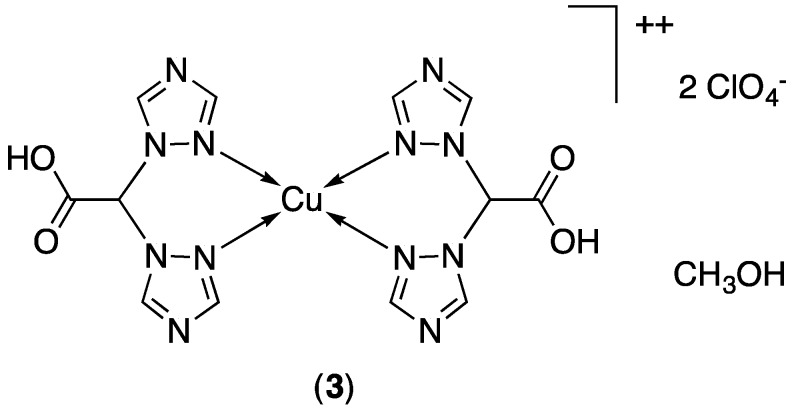
Proposed structure of compound **3**.

Following a simple and efficient synthetic methodology [59] the neutral ligand namely 2,2-di(1*H*-1,2,4-triazol-1-yl)acetic acid ([HC(COOH)(tz)_2_]) was prepared by an acid-base reaction, nucleophilic substitution and salification reaction, using 1,2,4-triazole (tz), dibromoacetic acid and sodium hydroxide at room temperature (Figure 2).

The related copper(II) complex [HC(COOH)(tz)_2_]_2_Cu(ClO_4_)_2_·CH_3_OH (**3**) was prepared at room temperature from the reaction of Cu(ClO_4_)_2_·6H_2_O with [HC(COOH)(tz)_2_] in methanol solution (Figure 2) with a stoichiometric ratio 2:1; an analogous product was been obtained in methanol solution with a stoichiometric ratio copper acceptor:ligand 1:1. Compound **3** was soluble in DMSO and air stable even as solutions. The authenticity of **3** was confirmed by elemental analysis, IR spectroscopy and Electrospray mass spectroscopy. The infrared spectrum shows all the bands required by the presence of the scorpionate donor. A broad absorption at 1664 cm^−1^, due to the carbonylic asymmetric stretching of the COOH groups, was slightly shifted with respect to the same absorption observed for the free ligand (1703 cm^−1^) being in accordance with the presence of the uncoordinated protonated carboxylic groups. The difference between asymmetric and symmetric frequencies Δ[(ν_asym_ COO) − (ν_sym_ COO)] was 299 cm^−1^ which supports the non-coordinating mode of the two carboxylic groups.

In the spectrum of compound **3,** weak absorptions due to the CH stretching have been observed in the range 2977–3134 cm^−1^, while a medium band at 1528 cm^−1^ was attributable to the ν(C=N_pz_) stretching vibrations. Strong peaks in the range 1083–1125 cm^−1^ were in accordance with the presence of the ClO_4_^−^ groups as counter-ion species.

Both positive- and negative-ion spectra of the complex **3**, dissolved in DMSO/CH_3_CN, were recorded at low voltage (3.5–4.0 kV). Under these experimental conditions the dissociation is minimal and most of the analyte is transported to the mass spectrometer as the intact molecular species [78]. The negative-ion spectrum of compound **3** was dominated by the fragments [ClO_4_]^−^ (*m*/*z* 99, 100), [HC(tz)_2_]^−^ (*m*/*z* 149, 10), [HC(COO)(tz)_2_]^−^ (*m*/*z* 193, 10), [Na(ClO_4_)_2_]^−^ (*m*/*z* 221, 100) and [Cu(ClO_4_)_3_]^−^ (*m*/*z* 360, 60). The ESI(+)-MS spectrum displayed a copper containing peak at *m*/*z* 290 ([{HC(COO)(tz)_2_}Cu(CH_3_OH)]^+^, 30%).

### 3.2. X-ray Crystallography

**Figure 3:**
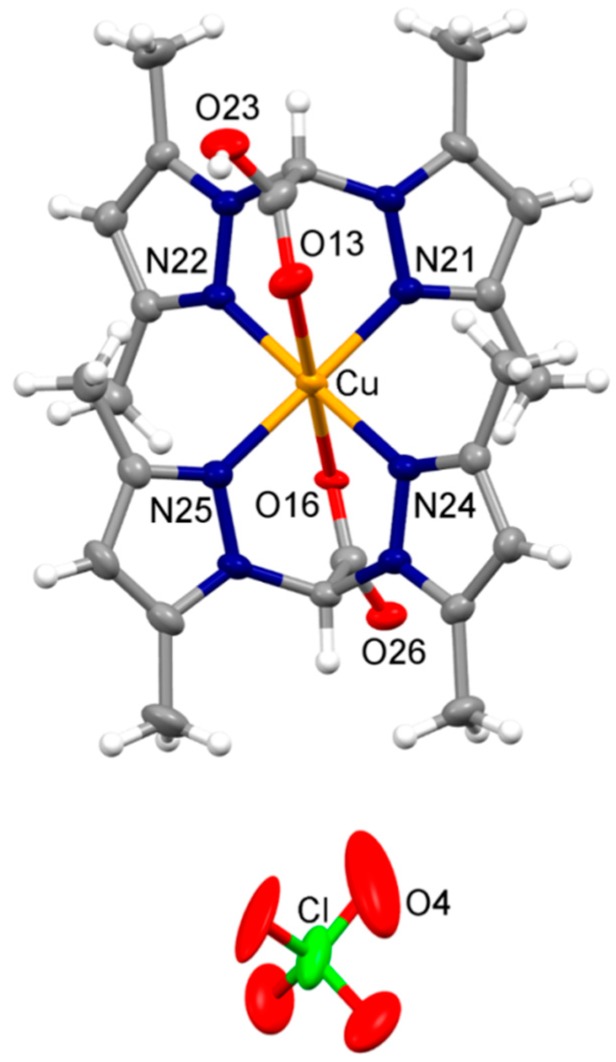
Molecular structure of (**1**) with thermal ellipsoids drawn at the 30% probability level. Disordered molecular fragments were removed for clarity.

The molecular structure of (**1**) is reported in Figure 3. The complex comprises two bis(pyrazolyl)methane moieties functionalized with a carboxylic function. Interestingly, only one of the two ligands was found deprotonated, whereas the second ligand presented the COOH function. The carboxylic and carboxylate functions were disordered in two positions, which were refined with site occupancy factors of 0.5 each (Figure S1, ESI), and the oxygen atom of the COO^−^ group of one ligand was closer to the metal center than the oxygen atom deriving from the protonated COOH. Overall, four nitrogen atoms of two ligands were located on an equatorial plane (Cu-N distances range 1.98–2.07 Å), an oxygen atom of the COO^−^ group occupied an apical position (Cu-O36/Cu-O36A, 2.32 and 2.36 Å, respectively), whereas the sixth position was occupied by the oxygen atom of the unprotonated carboxylic group (Cu-O13/O13A, 2.50 and 3.25 Å, respectively). This structural refinement implies that two geometries are adopted by the metal ion in the present structure. One structural image can be described as a distorted octahedron (Cu-O36 and Cu-O13 in apical position), and the second can be described as square pyramidal with Cu-O13 in apical position (Figure S2, ESI). The two different geometries were nonetheless in line with the electronic features of a d^9^ metal ion such as Cu^2+^. The positive charge of the complex was balanced by a severely disordered ClO_4_^−^ anion. The complex cations formed a supramolecular chain sustained by hydrogen bonds between the carboxylate and carboxylic functions of adjacent molecules (Figures S3 and S4, ESI). Each supramolecular chain was surrounded by anions arranged in a columnar structural site parallel to the *a* axis.

### 3.3. Biological Studies

#### 3.3.1. Activity in Monolayer and 3D Spheroid Cancer Cell Cultures

The in vitro antitumor activity of copper(II) complexes **1**–**3** and the uncoordinated ligands [HC(COOH)(pz^Me2^)_2_], [HC(COOH)(pz)_2_] and [HC(COOH)(tz)_2_] was evaluated against a panel of human cancer cell lines derived from solid tumors. Adherent cell lines representative of cervical (A431), pancreatic (BxPC3) colon (HCT-15), breast (MCF-7), lung (A549), and ovarian (2008) cancers, differently sensitive to cisplatin, were exposed to the indicated compounds for 72 h. IC_50_ values, calculated from the dose-response curves, are reported in Table 2.

Data analysis reveals that whereas the three uncoordinated ligands did not impact cell viability (average IC_50_ values were over 100 μM for all tested cell lines), copper(II) compounds elicited IC_50_ values in the micromolar range. Copper(II) complex **3**, bearing the bis(1,2,4-triazol-1-yl)-acetate ligand was noticeably less effective than cisplatin against all cancer cell lines, with IC_50_ values ranging between 16 and 70 μM. In contrast, both Cu(II) complexes **1** and **2**, obtained by coordinating the bis(pyrazol-1-yl)-acetate ligand with copper(II), displayed a similar growth inhibitory potency with mean IC_50_ values quite similar to those calculated for cisplatin (6.5, 8.9 and 7.1 μM for **1**, **2** and cisplatin, respectively). Noteworthy, against human lung A549 and human colon HCT-15 cancer cells, complex **1** was roughly 2-fold more effective than cisplatin. HCT-15 colon cancer cells are notoriously poorly chemosensitive to cisplatin. Hence, given the promising activity of both copper(II) derivatives **1** and **2** against HCT-15 cells, the in vitro antitumor activity of **1** and **2** was evaluated on a human colon cancer cell line pair which was selected for sensitivity/resistance to oxaliplatin (OXP), the key drug in FOLFOX (folinic acid, 5-fluorouracil, and OXP) combination chemotherapy for the management of colorectal cancers [79]. As for cisplatin, the clinical effectiveness of OXP is seriously hampered by the cancer cell resistance and, at present, few other drug regimens are available for patients with OXP-refractory colorectal cancers. The IC_50_ and resistance factor (RF) values calculated after 72 h of drug treatment by MTT test are listed in Table 3. Complex **3** confirmed itself as the least effective copper-based compound, eliciting IC_50_ values from 2 to 10 times higher than those calculated for OXP in resistant and sensitive colon cancer cells lines, respectively. Interestingly, complexes **1** and **2**, displayed a very similar cytotoxicity on both OXP-sensitive and -resistant cell lines, thus suggesting a different cross-resistance profile than OXP. The RF values (calculated by dividing IC_50_ for resistant cells by those for sensitive ones) were about 10 times lower than that of OXP, excluding the occurrence of cross-resistance phenomena.

The in vitro antitumor efficacy of the newly developed compounds was also tested on 3D colon cancer cell culture models. Differently from 2D monolayer culture, 3D spheroid cell culture systems comprise cancer cells in various cell growth stages. Consequently, the multicellular cancer spheroid model is recognized to better reflect the tumor mass in vivo regarding drug permeation, cell interactions, gene expression, hypoxia and nutrient gradients with respect to monolayer cell cultures [80]. Table 4 summarizes the IC_50_ values obtained after treatment of 3D cell spheroids of human HCT-15 colon cancer cells with copper(II) compounds as well as with cisplatin, as reference drug. In accordance with 2D chemosensitivity assays, complex **3** was scarcely effective, whereas complex **1** was as effective as cisplatin. Conversely, complex **2** was scarcely effective in 3D spheroids, eliciting IC_50_ values significantly higher than those calculated for cisplatin. We hypothesize that in this non-proliferative and very resistant tumor model the higher lipophilicity of **1**, due to the presence of methyl substituents on the pyrazolyl rings, might be responsible for the enhanced compound penetration in the core area of spheroids.

#### 3.3.2. Cellular Uptake Studies

**Figure 4:**
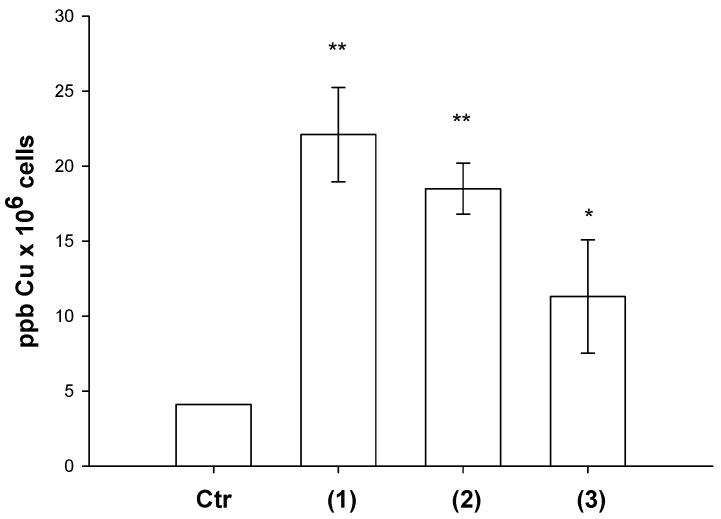
Intracellular copper content after treatment with compounds 1–3. LoVo cells were treated for 24 h with 2 μM of copper complexes, and intracellular copper amount was estimated by GF-AAS analysis. Error bars indicate the standard deviation. * *P* < 0.1, ** *P* < 0.01 compared with control.

With the purpose of underling a possible correlation between cellular uptake efficiency and cytotoxicity, LoVo cells were treated for 24 h with equimolar concentrations (2 μM) of copper(II) complexes and the intracellular copper amount was quantified by means of Graphite Furnace Atomic Absorption Spectrometry (GF-AAS) analysis. The results, expressed as ppb Cu per 10^6^ cells, are summarized in Figure 4. Among all, derivative **3** exhibited the lowest cellular uptake efficiency, in accordance with its low cytotoxicity. Among copper(II) complexes bearing the bis(pyrazol-1-yl)-acetate ligands, complex **1** was taken up slightly more efficiently than complex **2**, most likely as a result of its most pronounced lipophilic character. By comparing the cytotoxicity data with the cellular uptake results, a direct and linear correlation was demonstrated (R^2^ = 0.9). In fact, the intracellular copper content detected in LoVo cells follows the same cytotoxicity trend **1** > **2** > **3**.

#### 3.3.3. Oxidative Stress and Effects on the Ubiquitin-proteasome System

**Figure 5:**
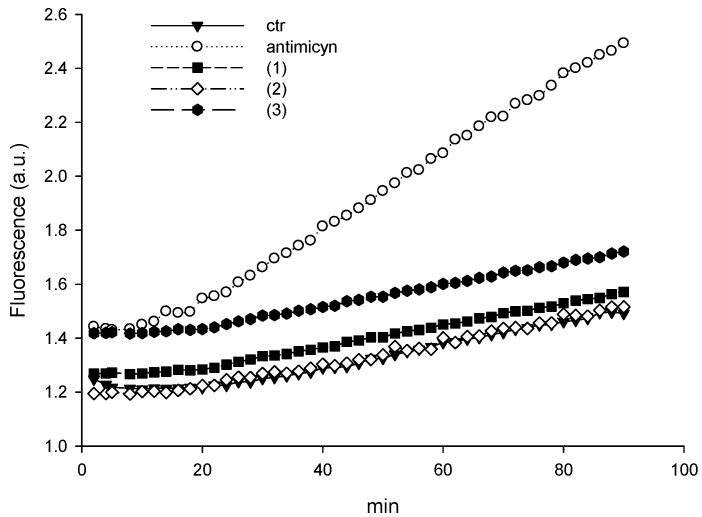
ROS production in LoVo cells. LoVo cells were initially loaded for 20 min at 37 °C with 10 mM CM-H_2_DCFDA in PBS. Subsequently, cells were treated with 15 μM of copper(II) complexes. Fluorescence intensity of DCFDA was detected.

It has been documented that oxidative stress and generation of ROS play a significant role in anticancer activity of copper(II) compounds [22,81]. To explore the molecular mechanisms underlying the cytotoxicity of newly synthetized copper(II) complexes, cellular production of ROS upon treatment with 15 μM of complex **1**, **2** or **3** was monitored in LoVo cells by using the peroxide-sensitive fluorescent probe CM-H_2_DCFDA (Figure 5). The oxidative stress inducer antimycin was included as positive control and markedly stimulated ROS formation. Conversely, for all copper complexes there was no ROS induction. Therefore, this result suggests that the cytotoxic effect promoted by the novel copper(II) complexes based on bis(azol-1-yl)-acetate heteroscorpionate ligands seems to be not connected with oxidative stress.

Since proteasome inhibition has emerged as a putative target for copper complexes, we also evaluated the effect of novel copper(II) complexes on the ubiquitin-proteasome system (UPS) [1].

**Figure 6:**
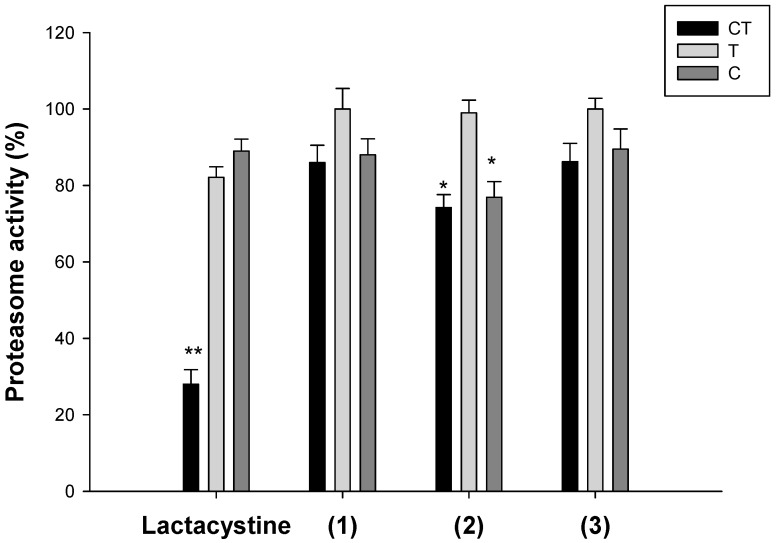
Proteasome inhibition. Inhibition of chymotrypsin-like (CT-L), trypsin-like (T-L), and caspase-like (C-L) activities of purified 20S proteasome was assessed fluorometrically after 60 min of incubation with increasing concentrations of compounds **1**–**3** or Lactacystin. * *P* < 0.1, ** *P* < 0.01 compared with control.

The ability of copper complexes to hamper the functioning of each individual proteasome active site, chymotrypsin-like (CT-L), trypsin-like (T-L), and caspase-like (C-L) activities was assessed in isolated 26S rabbit proteasomes after incubation with 15 μM of tested compounds. The results are shown in Figure 6. Lactacystin, an irreversible nonpeptidomimetic proteasome inhibitor, was used as a positive control. Our evaluation found that complexes **2** and **3** were able to affect CT-L and C-L activities, even if to a lesser extent than lactacystin. Conversely, no significant effects were recorded towards the T-L site. Even if the chymotrypsin-like sites are the major drug targets in cancer, it has been recently observed that co-targeting trypsin-like site increased cytotoxicity of copper complexes acting as proteasome inhibitors [13].

#### 3.3.4. TEM Analysis

**Figure 7:**
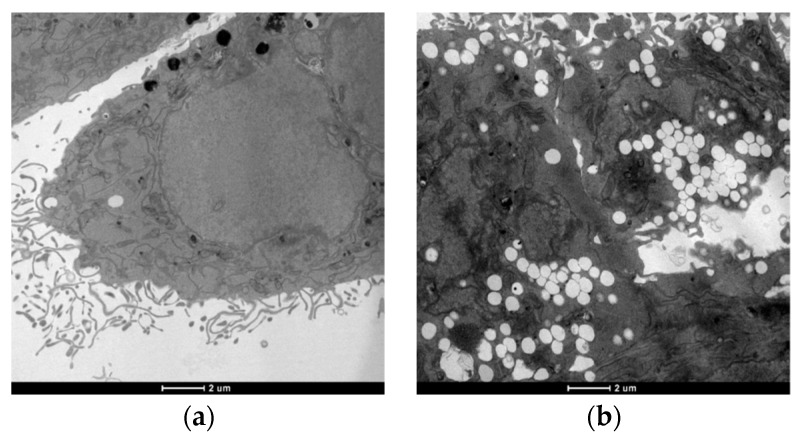
TEM analysis. LoVo cells after 24 h of treatment with (**a**) control; (**b**) IC_50_ of **1**.

Transmission electron microscopy (TEM) analysis has been performed in order to investigate the influence of copper compounds on LoVo cells morphology and their organelles. Colon cancer cells were exposed to IC_50_ of the most effective copper(II) derivative **1** for 24 h and then subjected to TEM analysis (Figure 7). 

Morphological analysis revealed no classical hallmarks of apoptosis, such as cell shrinkage, chromatin condensation or apoptotic bodies, suggesting a process of non-apoptotic cell death. Figure 7b clearly shows that complex **1**-treated LoVo cells underwent a massive cytoplasmic vacuolization in comparison with untreated cells (Figure 7a). Autophagy inhibitors, 3-MA (3-methyladenine) and monensin, did not reduce the presence of numerous vacuoles within the cytoplasm, induced by **1**, and vacuoles were not positive to monodansylcadaverine staining (data not shown), thus confirming the non-autophagic nature of **1**-induced cytoplasmic vacuolization. Therefore, all the findings derived by TEM analysis suggest that copper(II) complexes can trigger a process of paraptotic cancer cell death, an apoptosis alternative cancer cell death which was previously described for several classes of metal complexes and organic molecules [13,21,82].

This result is especially relevant in the context of the design of potential anticancer compounds be able to trigger alternative type of cell deaths in apoptosis-resistant cancer cells.

## 4. Conclusions

In our aim to find suitable ligands in the design and development of metal-based anticancer agents and encouraged by the promising in vitro and in vivo results obtained in recent anticancer screening of copper complexes, we focused our attention on the design and synthesis of bis(azol-1-yl)carboxylate heteroscorpionate ligands with pyrazole and 1,2,4-triazole rings. In particular, we synthesized and fully characterized the copper(II) complexes **1**–**3**: [HC(COOH)(pz^Me2^)_2_]Cu[HC(COO)(pz^Me2^)_2_]·ClO_4_, [HC(COOH)(pz)_2_]_2_Cu(ClO_4_)_2_ (pz^Me2^ = 3,5-dimethylpyrazole; pz = pyrazole) and [HC(COOH)(tz)_2_]_2_Cu(ClO_4_)_2_·CH_3_OH (tz = 1,2,4-triazole). The molecular structure of **1** was determined. The complex comprises two bis(pyrazolyl)methane moieties functionalized with a carboxylic function and interestingly, only one of the two ligands was found deprotonated, whereas the second ligand presents the COOH function. The in vitro antitumor activity of copper(II) complexes **1**–**3** and the uncoordinated ligands [HC(COOH)(pz^Me2^)_2_], [HC(COOH)(pz)_2_] and [HC(COOH)(tz)_2_] was evaluated against a panel of human cancer cell lines derived from solid tumors in monolayer and 3D spheroid cancer cell cultures with different Pt(II)-sensitivity. Whereas the three uncoordinated ligands did not impact cell viability, copper(II) complexes elicited IC_50_ values in the micromolar range. Complex **1** was active against cancer cell lines derived from solid tumors at low IC_50_ and this effect was retained in the spheroid model. For all copper complexes there was no ROS induction. Therefore, this result suggests that the cytotoxic effect promoted by the novel copper(II) complexes based on bis(azol-1-yl)-acetate heteroscorpionate ligands does not seem to be connected with oxidative stress. Since proteasome inhibition has emerged as a putative target for copper complexes, the effect of novel copper(II) complexes on the ubiquitin-proteasome system has been evaluated. Complexes **2** and **3** were able to affect CT-L and C-L activities, even if to a lesser extent than lactacystin. Conversely, no significant effects were recorded towards the T-L site. Structure and ultra-structure changes of treated cancer cells analyzed by TEM revealed the induction of a massive cytoplasmic vacuolization consistent with a paraptotic-like cancer cell death.

Even though further studies are needed to better clarify the mechanism of action triggered by these newly developed copper(II) complexes, overall these mechanistic and morphological studies suggested that they act by interfering with a protein/proteasome pathway, without affecting cellular redox homeostasis. More importantly, the ability of copper(II) complexes to induce paraptosis in cancer cells may provide a valuable strategy for overcoming resistance to apoptosis.

## Figures and Tables

**Table 1:** Summary of X-ray crystallographic data for (**1**).

Empirical formula	C_24_H_31_ClCuN_8_O_8_
Formula weight	658.56
Temperature/K	298
Crystal system	monoclinic
Space group	P2_1_/a
a/Å	13.831(2)
b/Å	16.048(2)
c/Å	14.198(2)
α/°	90
β/°	114.557(2)
γ/°	90
Volume/Å^3^	2866.3(7)
Z	4
ρ_calc_g/cm^3^	1.526
μ/mm^−1^	0.917
F(000)	1364.0
Crystal size/mm^3^	0.27 × 0.18 × 0.15
Radiation	MoKα (λ = 0.71073)
2θ range for data collection/°	3.154 to 51.362
Index ranges	−16 ≤ h ≤ 16, −19 ≤ k ≤ 19, −17 ≤ l ≤ 17
Reflections collected	32067
Independent reflections	5427 [R_int_ = 0.0546, R_sigma_ = 0.0344]
Data/restraints/parameters	5427/154/528
Goodness-of-fit on F^2^	1.027
Final R indexes [I ≥ 2σ (I)]	R_1_ = 0.0645, wR_2_ = 0.1669
Largest diff. peak/hole/e Å^−3^	1.50/−0.71

*R*1 = Σ||*F_o_*| − |*F_c_*||/Σ|*F_o_*|, *wR*2 = [Σ[*w*(*F_o_*^2^ − *F_c_*^2^)^2^]/Σ[*w*(*F_o_*^2^)^2^]]½, *w* = 1/[σ^2^(*F_o_*^2^) + (*aP*)^2^ + *bP*], where *P* = [max(*F_o_*^2^,0) + 2*F_c_*^2^]/3.

**Table 2:** Cytotoxic Activity.

	IC_50_ (µM) ± S.D.					
	A431	BxPC3	HCT-15	MCF-7	A549	2008
**(1)**	3.8 ± 1.1	2.5 ± 0.4	8.5 ± 0.6	10.5 ± 2.1	3.6 ± 0.5	10.8 ± 1.3
**(2)**	12.7 ± 1.2	7.5 ± 1.2	9.3 ± 2.7	10.2 ± 2.5	5.5 ± 0.2	7.9 ± 1.4
**(3)**	15.9 ± 5.8	18.5 ± 4.4	59.5 ± 2.7	39.6 ± 4.6	24.5 ± 1.9	69.3 ± 4.7
**[HC(COOH)(pz^Me2^)_2_]**	ND	ND	>100	>100	>100	>100
**[HC(COOH)(pz)_2_]**	ND	ND	>100	>100	>100	>100
**[HC(COOH)(tz)_2_]**	ND	ND	>100	>100	>100	>100
**Cisplatin**	1.7 ± 0.5	7.3 ± 1.2	15.3 ± 2.2	8.8 ± 1.4	7.5 ± 1.2	2.2 ± 1.0

Cells (3–8 × 10^3^ mL^−1^) were treated for 72 h with compounds. Cell viability was measured by means of MTT test. The IC_50_ values were calculated by 4-PL logistic model (*P* < 0.05). S.D. = standard deviation. ND = not detected.

**Table 3:** Cross-resistance profiles.

	IC_50_ (µM) ± S.D.		
	LoVo	LoVo-OXP	RF
**(1)**	4.2 ± 0.9	5.2 ± 0.6	1.2
**(2)**	4.0 ± 0.5	4.9 ± 0.6	1.2
**(3)**	15.3 ± 1.0	32.5 ± 1.0	2.1
**Oxaliplatin**	1.4 ± 0.7	15.2 ± 2.2	10.9

Cells (5 × 10^3^ mL^−1^) were treated for 72 h with tested compounds. Cell viability was measured by means of MTT test. IC_50_ values were calculated by 4-PL logistic model (*P* < 0.05). S.D. = standard deviation. RF = IC_50_ (resistant cells)/IC_50_ (wild-type cells).

**Table 4:** Cytotoxicity towards colon cancer cell spheroids.

	IC_50_ (µM) ± S.D.
	HCT-15
**(1)**	67.8 ± 14.9
**(2)**	107.8 ± 1.0
**(3)**	>100
**Cisplatin**	65.93 ± 3.85

Cancer cells spheroids (2.5 × 10^3^ cells/well) were treated for 72 h tested compounds. Cell viability was evaluated by means of the Acid Phosphatase APH test. IC_50_ values were calculated from the dose-response curves by 4-PL logistic model (*P* < 0.05). S.D. = standard deviation.

## Supplementary Materials

The following are available online, Figure S1: Asymmetric unit of (**1**) highlighting the disordered ClO_4_^−^ anion and the disordered carboxylic and carboxylate moieties, Figure S2: Distorted octahedral (above) and square pyramidal (below) geometries found in the structure, according to the disordered carboxylic/carboxylate groups, Figure S3: Portion of the crystal packing of (**1**) showing the weak interactions exchanged by the complex cation and the surrounding environment, Figure S4: Crystal packing of (**1**) as viewed along the *a* crystallographic axis.
