# Supplementary material for: Syntheses and Biological Studies of Cu(II) Complexes Bearing Bis(pyrazol-1-yl)- and Bis(triazol-1-yl)-acetato Heteroscorpionate Ligands
Source: Molecules. 2019 May 7;24(9):1761. doi: 10.3390/molecules24091761 (PMC6539868; doi:10.3390/molecules24091761)
Supplement: Supplementary file 1 [file molecules-24-01761-s001.pdf]

Supplementary Information (ESI)

Syntheses and biological studies of Cu(II) complexes bearing bis(pyrazol-1-yl)- and bis(triazol-1-yl)-acetato heteroscorpionate ligands

Maura Pellei,[a*] Valentina Gandin,[b*] Luciano Marchiò,[c] Cristina Marzano,[b] Luca Bagnarelli[a] and Carlo Santini[a]

[a]*School of Science and Technology, Chemistry Division, University of Camerino, via S. Agostino 1, 62032 Camerino, Macerata, Italy*
[b]*Department of Pharmaceutical and Pharmacological Sciences, University of Padova, via Marzolo 5, 35131 Padova, Italy*
[c]*Department of Chemistry, Life Science, and Environmental Sustainability, University of Parma, Parco Area delle Scienze 17A, Parma, 43124, Italy*

***Corresponding authors**:

(Maura Pellei) E-mail: maura.pellei@unicam.it

(Valentina Gandin) E-mail: valentina.gandin@unipd.it

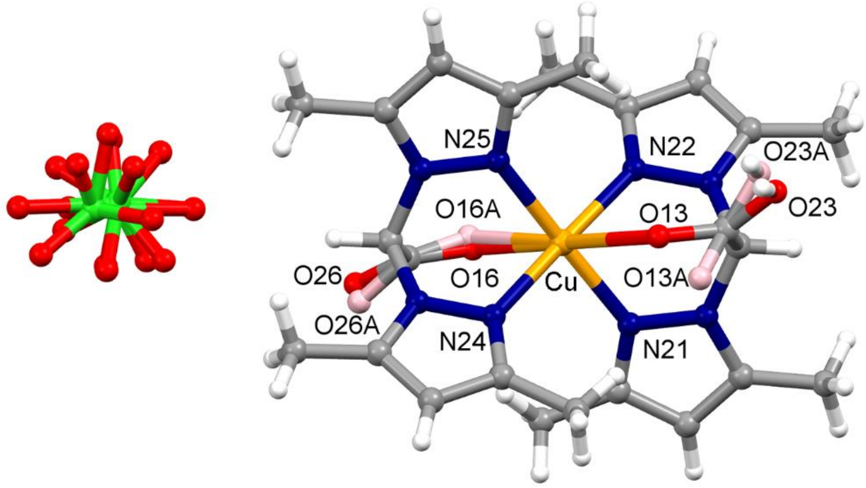

Figure S1. Asymmetric unit of (**1**) highlighting the disordered ClO_4^- anion and the disordered carboxylic and carboxylate moieties.

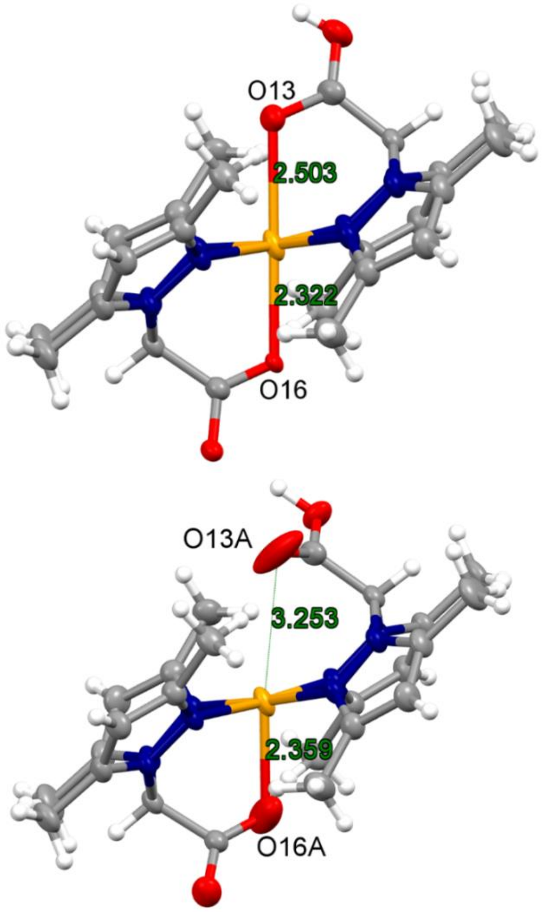

Figure S2. Distorted octahedral (above) and square pyramidal (below) geometries found in the structure, according to the disordered carboxylic/carboxylate groups.

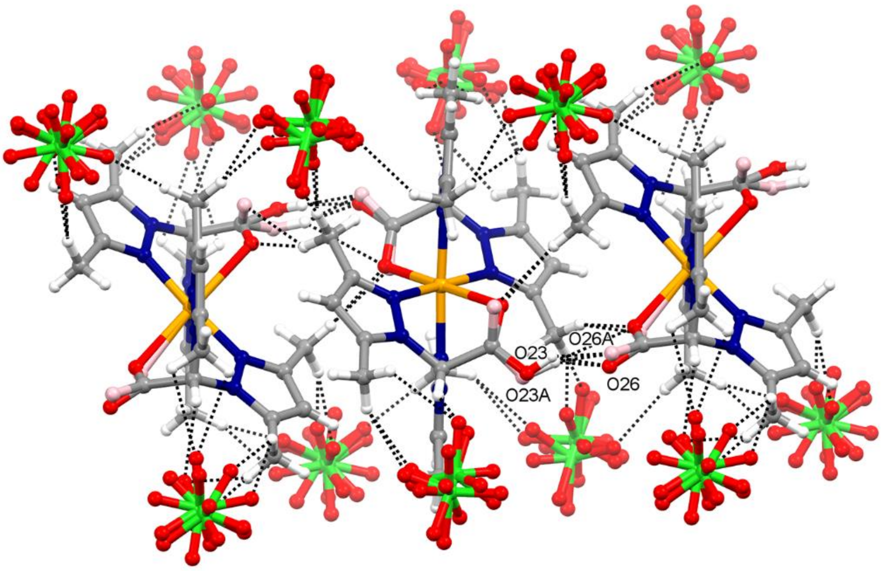

Figure S3. Portion of the crystal packing of (**1**) showing the weak interactions exchanged by the complex cation and the surrounding environment.

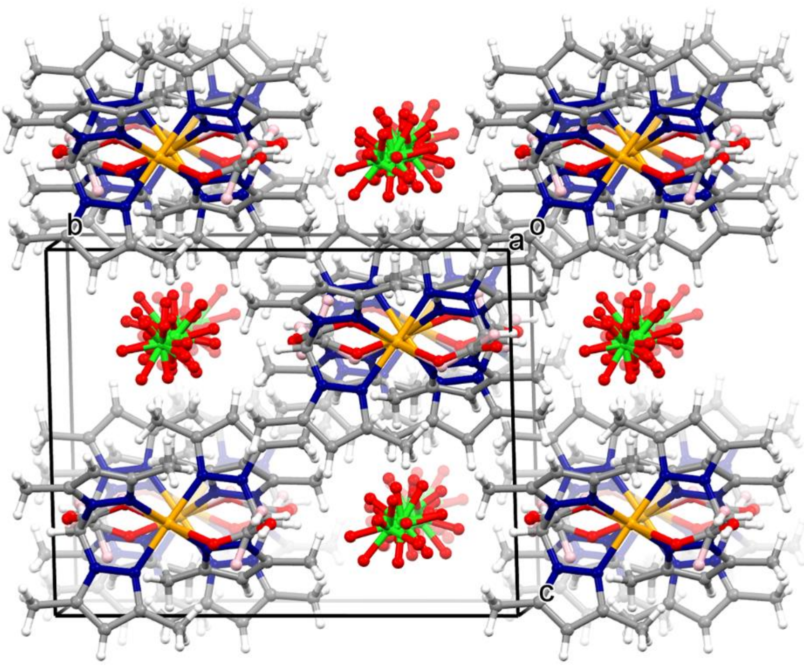

Figure S4. Crystal packing of (**1**) as viewed along the *a* crystallographic axis.
